# Vermin Riddance

**Published:** 1878-01

**Authors:** 


### VERMIN RIDDANCE.

HALF an ounce of soap boiled in a pint of water, and put on with a brush while boiling hot, infallibly destroys the bugs and their eggs.

*Flies* are driven out of a room by hanging up a bunch of the PLANTAIN, or *Fleawort* plant, after it has been dipped in milk.

*Rats and Mice* speedily disappear by mixing equal quantities of strong cheese and powdered *squills;* they devour this mixture with great greediness, while it is innocuous to man.

When it is remembered how many persons have lost their lives by swallowing, in mistake, mixtures of *strychnine*, *ratsbane*, *corrosive sublimate*, which are commonly employed for this purpose, it becomes a matter of humanity to publish these items.

*House Ants* ravenously devour the kernels of walnuts, and shellbarks or hickory nuts. Crack some of these, and place them on a plate near the infested places; and when the plate is full of the ants, throw the contents in the fire.

*Cockroaches*, as well as *Ants*, are driven away by strewing elderberry leaves on the shelves and other places frequented by these troublesome insects.

